# Music Therapy in Preterm Infants Reduces Maternal Distress

**DOI:** 10.3390/ijerph20010731

**Published:** 2022-12-30

**Authors:** Susann Kobus, Marlis Diezel, Monia Vanessa Dewan, Britta Huening, Anne-Kathrin Dathe, Peter B. Marschik, Ursula Felderhoff-Mueser, Nora Bruns

**Affiliations:** 1 Department of Paediatrics I, University Hospital Essen, University of Duisburg-Essen, 45147 Essen, Germany; 2 Centre for Translational Neuro- and Behavioural Sciences (C-TNBS), Faculty of Medicine, University Duisburg-Essen, 45147 Essen, Germany; 3 Center of Artistic Therapy, University Medicine Essen, 45147 Essen, Germany; 4 Department of Health and Nursing, Occupational Therapy, Ernst-Abbe-University of Applied Sciences Jena, 07745 Jena, Germany; 5 Child and Adolescent Psychiatry and Psychotherapy, University Medical Center Göttingen, 37075 Göttingen, Germany; 6 Leibniz ScienceCampus Primate Cognition, 37075 Göttingen, Germany; 7 iDN—Interdisciplinary Developmental Neuroscience, Division of Phoniatrics, Medical University of Graz, 8036 Graz, Austria; 8 Center of Neurodevelopmental Disorders (KIND), Center for Psychiatry Research, Department of Women’s and Children’s Health, Karolinska Institutet, 11330 Stockholm, Sweden

**Keywords:** preterm infants, maternal distress, neonatology

## Abstract

Preterm delivery is a stressful event for mothers, posing them at risk for post-traumatic stress reactions. This study examined the degree of depressive symptoms and post-traumatic stress in mothers of preterm infants born before 32 gestational weeks depending on whether the infant received music therapy in the neonatal intensive care unit (NICU) or not. We included 33 mothers of preterm infants enrolled in a previously described prospective randomized controlled trial, of whom 18 received music therapy (mean mothers’ age 34.1 ± 4.6 years) and 15 did not (mean mothers’ age 29.6 ± 4.2). The degree of depressive symptoms, anxiety and acute stress reactions of these mothers were measured by using the German version of the Center for Epidemiologic Studies Depression Scale (CES-D) and Impact of Events Scale-Revised (IES-R) one week after birth (T1) and at infants’ hospital discharge (T2). 605 music therapy sessions with a mean duration of 24.2 ± 8.6 min (range 10 to 50 min) were conducted two times a week from the second week of life (T1) until discharge (T2) to the infants from the intervention group. The infants from the control group received standard medical care without music therapy. The mean total CES-D score decreased from T1 (mean 34.7, 95% Confidence Interval (CI) 31.1–38.1) until T2 in all mothers (mean 16.3, 95% CI 12.6–20.1). Mothers whose infants received music therapy showed stronger declines of depressive and stress symptoms (with music therapy: CES-D mean difference of total score 25.7, 95% CI 20.0–31.3, IES-R mean difference of total score 1.7, 95% CI 0.9–2.5, IES-R mean difference of subcategory hyperarousal 10.2, 95% CI 6.2–14.3; without music therapy: CES-D mean difference of total score 9.5, 95% CI 3.8–15.3, IES-R mean difference of total score 0.1, 95% CI −1.0–1.2, IES-R mean difference of subcategory hyperarousal 1.6, 95% CI −4.7–7.9). Effect sizes were strong for CES-D, IES-R, and the hyperarousal subcategory, moderate for intrusion, and low for avoidance. These findings show that mothers of preterm infants are highly susceptible to supportive non-medical interventions such as music therapy to reduce psychological symptoms and distress during their infants’ NICU stay.

## 1. Introduction

More than every tenth baby worldwide is born before completing the 37th week of gestation, causing approximately 15 million preterm births every year [1]. To this, preterm birth is a major contributor to global neonatal mortality and morbidity [2]. From a psychological point of view, pregnancy and the transition to motherhood are considered a sequence of four characteristic mental states. In parallel with the intrauterine development of the child, the pregnant woman prepares for her future role as a mother. Premature delivery before 32 weeks of gestation disrupts the third of these phases, during which concretization of the 20th week of pregnancy takes place. For mothers of preterm infants born before 32 weeks of gestation, this phase is shortened and therefore they are unable to make these relevant adjustments in preparation for their child [3].

While the delivery of a child per se poses women at risk for post-traumatic stress reactions, anxiety, and depression, this effect is enhanced by preterm birth and the infant’s subsequent hospitalization in the Neonatal Intensive Care Unit (NICU) [2,4,5,6,7,8]. The not-yet anticipated mother role and the appearance of the preterm infant are the most important stressors for mothers in this context [9]. The physical separation from the infant, the physical and anthropometric condition and the differentiated appearance of the preterm infant compared to a full-term born influence parental feelings and bonding [10]. When their preterm infant is admitted to the NICU, mothers feel that they are borrowing their baby from staff they see as an expert caregivers and experience stress from losing their role as the infant’s primary caregiver [11]. This may exert a negative effect on mothers’ emotional state and perception of self-efficacy and be accompanied by feelings of being responsible for the preterm delivery [12,13,14,15].

Medical instability of the preterm infant, mechanical ventilation, and indwelling catheters complicate skin-to-skin contact between children and their mothers, which is essential for the establishment of mother-infant bonding and positively affects long-term outcomes after preterm birth [16,17]. Skin-to-skin contact between infants and their parents alleviates stress in parents and improves parental self-efficacy in handling the infant, getting involved in care, and feeling self-confident about caring for their infant [17]. Recent studies have focused on the impact of prematurity on early interactions between mothers and infants, finding that preterm dyads experience poorer and less synchronous interactions than full-term ones [18,19]. The oxytocin levels in mothers and their preterm infants are co-regulated by emotional connection or disconnection, and the autonomic co-conditioning learning mechanism can be used to transform a negative physiological and behavioral response between mother and infant into a positive one [20]. A randomized control study on family nurture interventions in the neonatal intensive care unit was able to show that promoting mother-child coordination through face-to-face communication in the first few months of life plays a key role in development [21].

Despite several studies on symptoms of depression and anxiety in mothers of preterm infants [18,19,22,23], the influence of music therapy on mothers’ psychological well-being has still not been extensively investigated [24,25,26]. Music therapy has been identified as having a positive impact on stress and anxiety in adults [27,28,29,30,31]. Parents of preterm infants who received family-centered music therapy felt positive about their own well-being and that of their infant [24]. Several studies have shown stabilizing effects of live music therapy on preterm infants’ vital signs [32,33,34]. Music therapy has also beneficial effects on vital signs when delivered to sleeping preterm infants [34].

The aim of this study was to assess the prevalence of maternal distress and well-being in mothers of preterm infants born < 32 weeks of gestation. We compared mothers whose babies received music therapy one week after birth and at discharge vs. ones who did not, hypothesizing music therapy to have an impact on maternal distress and well-being.

## 2. Methods

### 2.1. Study Design

Mothers of infants treated at the Neonatal Intensive Care Unit of the University Hospital Essen (Germany) who were participating in a randomized controlled trial (RCT) on music therapy in preterm infants < 32 weeks gestational age (German Clinical Trials Registry number: DRKS00025753) were prospectively surveyed one week after delivery (T1) and at the infant’s discharge (T2).

Mothers filled out surveys on symptoms of depression and anxiety using a depression scale (Allgemeine Depressions Skala lang, ADS-L, German version of the Center for Epidemiologic Studies Depression Scale, CES-D) [35] and the Impact of Event Scale revised questionnaire (IES-R) [36] one week after birth (T1) and at the infants’ discharge from hospital (T2). The surveys were added to the main study protocol in January 2020, 15 months after the start of the study, because informal unstandardized questionnaires in the first phase of the RCT suggested parental benefit from music therapy [24].

### 2.2. Infant Eligibility and Recruitment for the Main RCT

Infants born between October 2018 and May 2021 at the University Hospital Essen < 32 weeks gestational age were eligible for participation in the main RCT. If they were known at the time of screening for study participation, congenital hearing disorders, intraventricular hemorrhage grade III after papile, periventricular infarction, and cerebral malformations were exclusion criteria for this study. At a minimum age of 72 h, the parents’ declaration of consent was signed during the first week of life. The local ethics committee of the medical faculty of the University of Duisburg-Essen approved this study (18-8035-BO).

### 2.3. Randomization and Music Therapy Intervention

Eighty clinically stable preterm infants were recruited and randomized 1:1 to either the intervention or the control group. Infants of the intervention group received live performed music therapy from a qualified music therapist. The first session was performed during the second week of life and from then on was provided twice per week until discharge as previously described [24,34,37]. Depending on the clinical condition of the child and the presence of the parents, the music therapy was provided when the child was in the incubator, in a warming bed or on the parents’ arms, legs or shoulders. If the infant was too unstable to be removed from the cot or incubator the parents touched their infant with their hand during the session. Each session was provided individually for each patient and the therapist used her singing at low volume or played soft sounds of the instrument sansula [24,34,38]. The presence of the parents was documented after each intervention.

### 2.4. Outcomes

The outcomes were CES-D, IES-R scores, and IES-R subscales at discharge.

### 2.5. Questionnaires

#### 2.5.1. CES-D

The Allgemeine Depressions Skala (ADS-L) is the German version of the Center for Epidemiologic Studies Depression Scale (CES-D) and contains twenty items addressing depressive symptoms [39]. The CES-D includes self-descriptive first-person statements with reference to the week before the assessment. The items ask about the frequency of affective, cognitive, somatic and social symptoms that typically occur in depression. Respondents indicate how often they experienced those symptoms within the last week on a 4-point Likert scale: “rarely or none of the time (less than one day)” (0); “some or a little of the time (one to two days)” (1); “occasionally or a moderate amount of time (three to four days)” (2); or “most or all of the time (five to seven days)” (3). The resulting score ranges from 0 to 60. Four items (Nos. 4, 8, 12, 16) are scaled inversely and serve as criteria for the validity of the answers. It is a control of a correct understanding and exclusion of misrepresentations. Two content-based subscales can be used. Items 1, 3, 4, 6, 8–10, 12–16, 18 and 19 refer to mental symptoms whereas items 2, 5, 7, 11, 17 and 20 refer to somatic symptoms.

#### 2.5.2. IES-R

Weiss and Marmer (1996) [40] extended the original version of the IES which was originally derived from the theoretical model traumatic stress consequences [41]. The items of the new subscale hyperarousal were obtained from clinical observations of post-traumatic stress disorder. The German-language version of the IES-R was obtained through a translation back-translation process.

The IES-R has proven valid to differentiate subjects with acute stress from subjects with post-traumatic stress [42]. The IES-R is a self-reported, 22-item questionnaire based on three clusters of symptoms identified in the Diagnostic and Statistical Manual of Mental Disorders, third edition (DSM-III), as indicators of post-traumatic stress disorder (PTSD). It provides a structured way for the patient to communicate distress as a response to a traumatic event when she or he may not have the words to say it [43].

The three subscales “Intrusion”, “Avoidance” and “Hyperarousal” show typical kinds of individual reactions or symptoms to extremely stressful events. Intrusion is assessed with eight items on the scale (1, 2, 3, 6, 9, 14, 16, 20), Avoidance with eight items (5, 7, 8, 11, 12, 13, 17, 22) and Hyperarousal with six items (4, 10, 15, 18, 19, 21). For the subscales intrusion and hyperarousal arise maximum total scores of 35 and for the avoidance subscale 40. A PTSD diagnosis can be made using the following formula [41]:

Diagnostic value X = −0.02 ∗ Intrusion + 0.07 ∗ Avoidance + 0.15 ∗ Hyperarousal − 4.36

If the resulting value is >0.0, a PTSD diagnosis is likely. The formula was cross-validated in two traumatization groups. The sensitivity for the PTSD diagnosis is 0.70 or 0.76, the specificity is 0.88 or 0.90.

The target group for this questionnaire is people with extreme experiences such as sexualized acts of violence, experiences of war, and experienced natural disasters. Experiencing life-threatening illnesses, accidents or the event of extremely preterm birth before the completion of the 32nd week of pregnancy support the use of the questionnaire.

### 2.6. Intervention

Music therapy was performed two times per week in clinically stable infants from the second week of life until discharge. During the music therapy sessions, the infants remained in the incubator, heated bed, bed or in physical contact with the mother or father. The music therapist played the instrument sansula at a low volume or sang improvised melodies, based on the individual reactions and synchronized with the breathing of the infants. The sansula creates a long-lasting and soft sound.

### 2.7. Statistical Analyses

Continuous variables are presented as median with interquartile range (IQR) if skewed and mean with confidence intervals (CI) if normally distributed. Discrete variables are presented as counts and relative frequencies. SAS Enterprise Guide 8.4 (SAS Institute Inc., Cary, NC, USA) was used to perform statistical analyses and produce figures. Effect sizes to compare mean differences (T1 − T2) between the control and intervention groups were calculated according to Cohen using pooled weighted standard deviations.

## 3. Results

### 3.1. Patients

**Figure 1:**
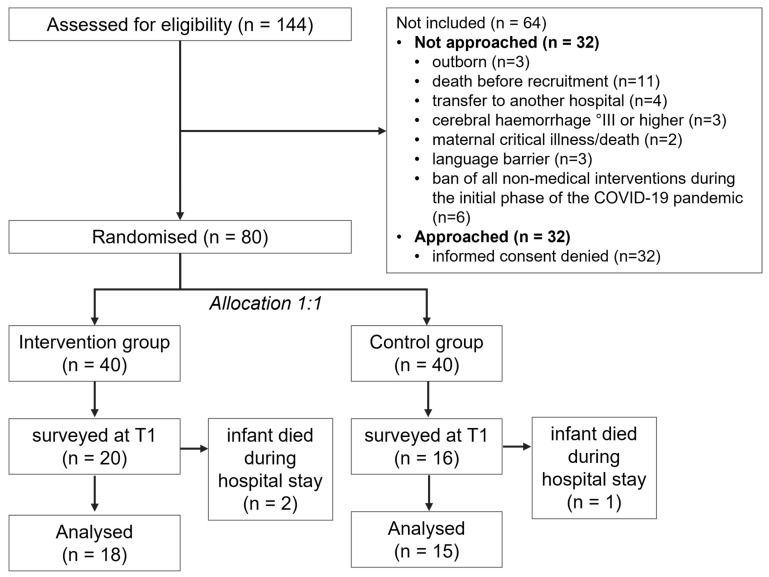
Flow chart of the included and excluded participants of the study.

During the study period, 144 premature infants were born before 32 weeks gestation, of which 64 were not included (Figure 1) and 40 each were randomized to either the intervention or the control group. At the introduction of the questionnaire assessed for this manuscript, 44 of the total 80 infants had already been recruited. The mothers of the latter 36 preterm infants (20 intervention group and 16 control group) were surveyed prospectively. Three mothers of these did not submit questionnaires (2 intervention groups and 1 control group) because their infants died during the hospital stay. The sociodemographic characteristics of the 33 mothers who completed the questionnaires at T1 and at T2 are presented in Table 1.

605 music therapy sessions were conducted in the intervention group between corrected gestational ages of 24 + 5 and 43 + 5 weeks. The mean duration of each music therapy session was 24.2 ± 8.6 min (range 10 to 50 min). Parents were present in 252 (42%) sessions. Ninety-three music therapy interventions were conducted with physical contact with the mother. In 43 interventions was physical contact with the father. 96 sessions were performed with infants in the heated bed, 186 sessions in a non-heated bed and 187 sessions in the incubator.

### 3.2. CES-D and IES-R

**Figure 2:**
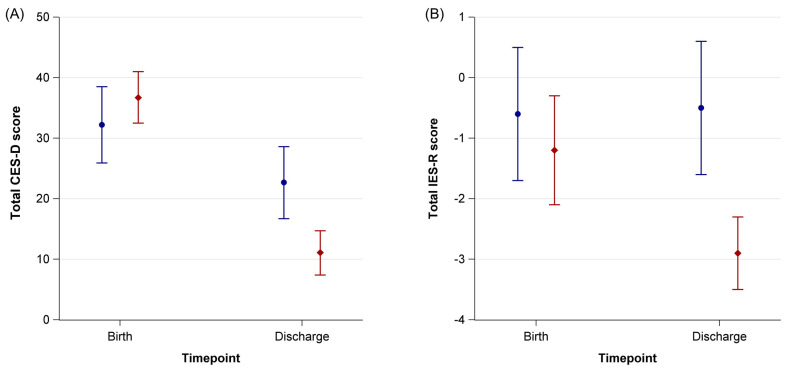
Total CES-D scores (**A**) and total IES-R scores (**B**) of mothers of preterm infants born < 32 gestational weeks with music therapy (red diamonds) and without music therapy (blue circles) one week after birth and at the infants’ discharge from hospital. Error bars represent 95% confidence intervals.

In the overall cohort, the mean CES-D total score dropped from 34.7 (95% CI 31.1–38.2) at T1 to 16.3 (95% CI 12.6–20.1) at T2 of the hospital. The mean difference was −18.3 (95% CI −23.1–(−13.5)). At baseline one T1, CES-D scores did not differ between mothers whose infants received music therapy and mothers whose infants did not receive music therapy (36.7 (95% CI 32.5–41.0) vs. 32.2 (25.9–38.5)). At T2, mothers in the music intervention group had lower CES-D scores compared to mothers in the control group (11.1 (7.4–14.7) vs. 22.7 (16.7–28.6)) (Table 2 and Table S1, Figure 2).

The mean IES-R total score was −0.9 (95% CI −1.6–(−0.3)) at T1 and −1.8 (95% CI −2.5–(−1.1)) at T2 of the hospital. The mean difference was −0.9 (95% CI −1.6–(−0.2)).

**Figure 3:**
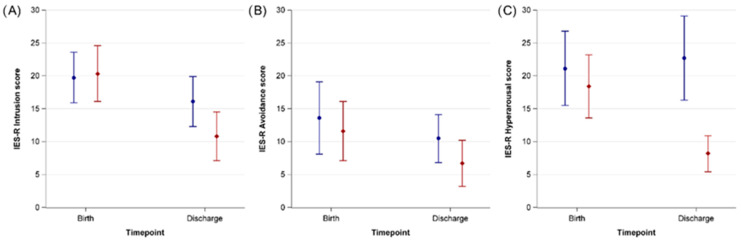
Absolute changes of IES-R Items of mothers of preterm infants with music therapy (red diamonds) and without music therapy (blue circles) one week after birth and at the infants’ discharge from hospital. (**A**) Intrusion. (**B**) Avoidance. (**C**) Hyperarousal. Error bars represent 95% confidence intervals.

Table S2 shows the means of the absolute values of IES-R items for mothers’ respondents, whose infants received music therapy or not, at T1 and at T2 of the infant from hospital. When analyzing the total score and the subgroups intrusion, avoidance and hyperarousal we saw a greater difference in the intervention group between T1 and T2 (Table 3 and Figure 3).

### 3.3. Mean Differences of CES-D and IES-R Total Scores

The mean differences of CES-D total scores between baseline and discharge were −25.7 (95% CI −31.3–(−20.0)) in mothers with music therapy and −9.5 (95% CI −15.3–(−3.8)) in mothers without music therapy (Figure S1). There are mean differences in IES-R total scores between baseline and discharge in the IES-R as well as in the CES-D. The mean differences were −1.7 (−2.5–(−0.9)) in mothers with music therapy and 0.1 (−1.0–1.2) in mothers without music therapy (Figure S1). When analyzing the subcategories intrusion, avoidance and hyperarousal we saw the smallest differences in avoidance (Figure S2). The effect sizes were strong for CES-D, IES-R, and the hyperarousal subcategory, moderate for intrusion, and low for avoidance (Table 3).

## 4. Discussion

This study found a markedly stronger decrease in depressive symptoms, measured as total CES-D scores, in preterm infants’ mothers from birth until their infant’s hospital discharge if the infant received music therapy. The perceived distress from premature delivery, measured as total IES-R scores, decreased in mothers in the intervention group whereas the scores remained stable in the control group. Among the IES-R subscales, the strongest difference was observed for hyperarousal with a slight increase in the non-intervention group and a strong decrease in the intervention group.

Our results show that music therapy, primarily intended to promote physiological stability and potentially improve neurodevelopmental outcomes of preterm infants [44], reduces symptoms of depression and distress in mothers [45,46,47]. Mothers of preterm infants are at higher risk for anxiety, depression, and post-traumatic stress disorder compared to term-born neonates’ mothers. They are unprepared for parenthood and experience a broken connection with the infant [12,48]. The long stay in the intensive care unit after preterm birth is an additional stressful experience for parents of preterm infants [10].

In the context of other diseases such as cancer, sickle cell disease or chronic pain, music therapy has been identified as a potent modulator of stress and anxiety in adults [27,28,29,30,31]. Music therapy is effective as supportive therapy in cancer patients during treatment to attenuate fatigue, anxiety and depression [27,28]. Music therapy can also be a fundamental component of rehabilitation programs to promote well-being, and improve physical and emotional well-being and quality of life in adults with chronic disease [27,29,30]. 

While the primary purpose of the NICU is to provide life-saving medical assistance to the infants, it is part of building the mother-child interaction already in the delivery room and the early dynamics of their relationship [49]. Differences in maternal interaction behavior between mothers of preterm and term-born infants are most pronounced in the first six months after birth [50,51]. Infant brain development is tied to autonomic co-conditioning mechanisms and one’s ability to understand and empathize with what others think and feel depends on early autonomy socio-emotional learning between mother and infant that begins in utero and continues after birth [52]. To overcome these differences, preterm infants’ parents are encouraged to take an active part in the care of their infant already during the NICU stay, including the promotion of close skin-to-skin contact known as kangaroo care [50,53]. A family-centered care program in the inpatient setting with coordinated aftercare increases parental satisfaction, shortens the length of hospital stay and is, therefore, a viable concept [54]. While these measures have been proven effective, the results of our study highlight the susceptibility of mothers to music therapy as an additional stress-reducing intervention in the NICU. Thus, the effect of music therapy seems to extend beyond the intended benefits for the preterm infant by positively affecting mothers, as well. 

Music therapy as a simple, safe, and side-effect-free intervention empowers maternal competence to interact with the baby and facilitate synchrony, which may act as a protective factor for the infant’s development, maternal well-being, and mother-infant bonding [17] and reinforces the Calming Cycle Theory about the emotional relationship between mother and child, providing opportunities for positive intervention when problems arise [20].

A major limitation of our study is the fact that the questionnaires rely on subjective assessments, which are often associated with negatively altered post-traumatic cognitions. No diagnoses of depression or anxiety could be made from the questionnaires, as they are primarily designed to assess the degree of symptoms [55]. Well-being is not measured at CES-D as comprehensively as in established scales designed to assess well-being [56]. Not all criteria for emotional numbness listed in the Diagnostic and Statistical Manual of Mental Disorders are covered by the questionnaires we used. Further, the small sample size did not allow detailed subgroup analyses, to assess differences in the effects of the intervention, e.g., between families with or without the background of migration, different gestational ages or educational backgrounds. It seems reasonable that the strongest effects may be observed in the most burdened mothers, therefore detailed assessment of the psychosocial background would be desirable for future studies that focus on the effect of preterm infants’ music therapy on parental well-being. Another factor that might have influenced mothers’ stress levels is the time spent with their infants in the NICU, which was not measured in this study. Future studies should also evaluate the interaction between mothers and infants and if music therapy might also influence the emotional connection.

## 5. Conclusions

In summary, this study provides evidence that music therapy care for preterm infants in an inpatient setting exerts positive effects not only on preterm infants themselves but also on their mothers’ levels of depression and distress. As mothers and their infants are inextricably intertwined in their autonomic co-regulation and bonding, the effect of music therapy as a stress-reducing intervention may reach far beyond a transient stabilization of the infant’s vital signs, possibly helping to unleash and maintain a positive cycle of reduced stress and improved bonding. Therefore, medical and economic considerations on the effectiveness of music therapy as part of family-centered care in the NICU must imperatively include effects on the maternal psyche as well.

## Figures and Tables

**Table 1:** Sociodemographic characteristics of mothers in the intervention group and the control group.

	Intervention Group	Control Group
Mothers [n (%)]	18 (55%)	15 (45%)
Mother’s age at delivery (years) [mean ± SD (range)]	34.1 ± 4.6 (25.0–40.0)	29.6 ± 4.2 (20.0–36.0)
Education		
High (13 years of education completed) [n (%)]	6 (33%)	3 (20%)
Middle (9 years of education completed) [n (%)]	7 (39%)	6 (40%)
Low (8 years of education completed) [n (%)]	2 (11%)	2 (13%)
Other degree [n (%)]	1 (6%)	0 (0%)
No degree [n (%)]	2 (11%)	2 (13%)
Not specified [n (%)]	0 (0%)	2 (13%)
Gravida [mean ± SD (range)]	2.9 ± 2.2 (1.0–8.0)	2.6 ± 2.0 (1.0–8.0)
Primagravida [n (%)]	8 (44%)	5 (33%)
Multigravida [n (%)]	10 (56%)	10 (67%)
Number of current child [mean ± SD (range)]	2.4 ± 1.7 (1.0–6.0)	2.3 ± 1.9 (1.0–8.0)
GA (weeks) [mean ± SD (range)]	29.1 ± 2.4 (23.9–31.7)	28.7 ± 2.5 (22.9–31.7)
≤25 weeks [n (%)]	2 (11%)	3 (20%)
26, 27 weeks [n (%)]	4 (22%)	3 (20%)
28, 29 weeks [n (%)]	2 (11%)	3 (20%)
30, 31 weeks [n (%)]	10 (56%)	6 (40%)
Birth mode		
Primary cesarean section [n (%)]	9 (50%)	7 (47%)
Secondary cesarean section [n (%)]	2 (11%)	1 (7%)
Emergency cesarean section [n (%)]	3 (17%)	4 (27%)
Spontaneous [n (%)]	4 (22%)	3 (20%)

**Table 2:** Total CES-D scores and total and subcategories IES-R scores (95% confidence interval) of mothers of preterm infants with and without music therapy one week after birth (T1) and at the infants’ discharge from hospital (T2).

	T1		T2	
	Intervention Group	Control Group	Intervention Group	Control Group
CES-D Total Score	36.7 (32.5–41.0)	32.2 (25.9–38.5)	11.1 (7.4–14.7)	22.7 (16.7–28.6)
IES-R Intrusion	20.3 (16.1–24.6)	19.7 (15.9–23.6)	10.8 (7.1–14.5)	16.1 (12.3–19.9)
IES-R Avoidance	11.6 (7.1–16.1)	13.6 (8.1–19.1)	6.7 (3.2–10.2)	10.5 (6.8–14.1)
IES-R Hyperarousal	18.4 (13.6–23.2)	21.1 (15.5–26.8)	8.2 (5.4–10.9)	22.7 (16.3–29.1)
IES-R Total Score	−1.2 (−2.1–(−0.3))	−0.6 (−1.7–0.5)	−2.9 (−3.5–(−2.3))	−0.5 (−1.6–0.6)

All data are presented as mean and 95% confidence intervals. T1 = one week after birth. T2 = at infants’ hospital discharge.

**Table 3:** Mean differences of the total CES-D Scores and the total and subcategories IES-R Scores of mothers of preterm infants born < 32 gestational weeks with and without music therapy between assessment one week after birth and the infant’s discharge.

	Difference between T1 and T2 Mean (95% Confidence Interval)		
	Intervention Group	Control Group	Effect Size *
Total Score CES-D	−25.7 (−31.3–(−20.0))	−9.5 (−15.3–(−3.8))	1.5
IES-R Intrusion	−9.5 (−13.7–(−5.3))	−3.7 (−7.9–0.6)	0.7
IES-R Avoidance	−4.9 (−9.3–(−0.5))	−3.1 (−8.1–1.8)	0.2
IES-R Hyperarousal	−10.2 (−14.3–(−6.2))	1.6 (−4.7–7.9)	1.2
Total Score IES-R	−1.7 (−2.5–(−0.9))	0.1 (−1.0–1.2)	1.1

T1 = one week after birth. T2 = at infants’ hospital discharge. * Cohen’s d.

## Data Availability

Original data will be made available to any qualified researcher upon request.

## Supplementary Materials

The following supporting information can be downloaded at: https://www.mdpi.com/article/10.3390/ijerph20010731/s1, Figure S1: Mean differences of the total CES-D Scores and the total IES-R Scores of mothers of preterm infants born < 32 gestational weeks with and without music therapy between assessment one week after birth and the infant’s discharge. (A) CES-D. (B) IES-R. Error bars represent 95% confidence intervals.; Figure S2: Mean differences of IES-R Items between mothers of preterm infant born < 32 gestational weeks with and without music therapy between assessment one week after birth and the infant’s discharge. (A) Intrusion. (B) Avoidance. (C) Hyperarousal.; Table S1: Mean of absolute values (95% confidence interval) of CES-D Items of mothers of preterm infants with and without music therapy one week after birth (T1) and at the infants’ discharge from hospital (T2); Table S2: Mean of absolute values (95% confidence interval) of IES-R Items of mothers of preterm infants with and without music therapy one week after birth (T1) and at the infants’ discharge from hospital (T2).
