# Supplementary material for: Music Therapy in Preterm Infants Reduces Maternal Distress
Source: Int J Environ Res Public Health. 2022 Dec 30;20(1):731. doi: 10.3390/ijerph20010731 (PMC9819311; doi:10.3390/ijerph20010731)
Supplement: Supplementary file 1 [file ijerph-20-00731-s001.zip › ijerph-2086757-supplementary.pdf]

Supplementary Materials

Table S1. Mean of absolute values (95% confidence interval) of CES-D Items of mothers of preterm infants with and without music therapy one week after birth (T1) and at the infants' discharge from hospital (T2).

	Intervention	Group	Control Group	
	**T1 (95% CI)**	**T2 (95% CI)**	**T1 (95% CI)**	**T2 (95% CI)**
CES-D 1 … I was bothered by things.	2.2 (1.7 - 2.7)	0.8 (0.5 - 1.1)	2.1 (1.5 - 2.6)	1.5 (1.1 - 1.9)
CES-D 2 … I had a poor appetite.	2.1 (1.5 - 2.6)	0.2 (-0.1 - 0.5)	1.5 (0.8 - 2.1)	1.1 (0.6 - 1.5)
CES-D 3 … I could not shake off the blues.	1.9 (1.5 - 2.4)	0.3 (0.0 - 0.5)	1.7 (1.2 - 2.3)	0.6 0.3 - 0.9)
CES-D 4 … I felt as good as other people.	2.5 (2.1 - 2.9)	1.4 (0.9 - 1.9)	1.7 (1.0 - 2.3)	1.9 (1.2 - 2.6)
CES-D 5 … troubles keeping my mind on what I was doing.	2.3 (1.9 - 2.6)	0.7 (0.3 - 1.1)	1.7 (1.1 - 2.4)	1.3 (0.9 - 1.7)
CES-D 6 … I felt depressed.	2.0 (1.6 - 2.4)	0.2 (0.0 - 0.4)	1.9 (1.5 - 2.4)	0.7 (0.3 - 1.1)
CES-D 7 ... everything was an effort for me.	1.9 (1.4 - 2.5)	0.7 (0.3 - 1.1)	2.2 (1.7 - 2.7)	1.4 (1.0 - 1.8)
CES-D 8 … I felt hopeful about the future.	1.9 (1.5 - 2.3)	0.6 (0.0 - 0.4)	1.3 (0.6 - 1.9)	1.3 (0.8 - 1.8)
CES-D 9 … I thought my life is a failure.	0.7 (0.2 - 1.1)	0.0	0.5 (0.0 - 1.0)	0.1 (-0.1 - 0.2)
CES-D 10 … I felt fearful.	2.7 (2.3 - 3.0)	1.0 (0.7 - 1.3)	2.1 (1.5 - 2.8)	1.6 (1.1 - 2.1)
CES-D 11 … my sleep was restless.	1.8 (1.3 - 2.3)	0.4 (0.1 - 0.8)	2.1 (1.7 - 2.6)	1.4 (1.0 - 1.8)
CES-D 12 … I was happy.	2.6 (2.2 - 2.9)	0.8 (0.3 - 1.2)	2.1 (1.6 - 2.6)	1.7 (1.2 - 2.3)
CES-D 13 … I talked less than usual.	1.4 (1.0 - 1.7)	0.3 (0.0 - 0.6)	1.3 (0.6 - 1.9)	0.7 0.3 - 1.1)
CES-D 14 … I felt lonely.	1.4 (0.9 - 1.9)	0.2 (-0.1 - 0.5)	1.4 (0.7 - 2.1)	1.1 (0.7 - 1.6)
CES-D 15 … people were unfriendly to me.	0.2 (-0.1 - 0.3)	0.0	0.3 (-0.1 - 0.7)	0.2 (-0.1 - 0.5)
CES-D 16 … I enjoyed the life.	2.8 (2.6 - 3.0)	1.7 (1.1 - 2.3)	2.9 (2.7 - 3.1)	2.6 (2.2 - 3.0)
CES-D 17 … I had to cry.	2.3 (2.0 - 2.7)	0.8 (0.3 - 1.3)	2.2 (1.8 - 2.6)	1.2 (0.7 - 1.7)
CES-D 18 … I felt sad.	2.3 (1.9 - 2.8)	0.5 (0.2 - 0.8)	2.0 (1.4 - 2.6)	1.0 (0.6 - 1.4)
CES-D 19 … I felt people disliked me.	0.1 (0.0 - 0.3)	0.1 (0.0 - 0.3)	0.3 (-0.1 - 0.6)	0.3 (0.0 - 0.7)
CES-D 20 … I could not get going.	1.6 (1.1 - 2.1)	0.3 (0.0 - 0.6)	0.9 (0.5 - 1.4)	0.7 (0.5 - 1.0)

CI = confidence interval. T1 = one week after birth. T2 = at infants' hospital discharge.

Table S2. Mean of absolute values (95% confidence interval) of IES-R Items of mothers of preterm infants with and without music therapy one week after birth (T1) and at the infants' discharge from hospital (T2)

	Intervention Group		Control Group	
	T1 (95% CI)	T2 (95% CI)	T2 (95% CI)	T2 (95% CI)
IES-R 1 … any reminder brought back feelings about it.	4.3 (3.7 - 5.0)	2.7 (1.8 - 3.5)	4.1 (3.2 - 5.0)	3.1 (2.4 - 3.9)
IES-R 2 … I had troubles staying asleep.	2.9 (2.0 - 3.9)	1.6 (0.7 - 2.5)	4.0 (3.2 - 4.8)	4.0 (3.2 - 4.8)
IES-R 3 … other things kept making me think about it.	2.8 (2.1 - 3.5)	2.0 (1.2 - 2.8)	3.3 (2.6 - 4.0)	2.6 (1.7 - 3.5)
IES-R 4 … I felt irritable and angry.	2.1 (1.2 - 2.9)	0.7 (0.1 - 1.2)	2.3 (1.3 - 3.2)	1.8 (0.9 - 2.7)
IES-R 5 … I avoided letting myself get upset when I thought about it or was reminded of it.	1.7 (1.1 - 2.3)	0.9 (0.3 - 1.5)	1.8 (1.1 - 2.5)	1.9 (1.1 - 2.6)
IES-R 6 … I thought about it when I didn`t mean to.	3.3 (2.4 - 4.2)	1.4 (0.8 - 2.1)	3.4 (2.7 - 4.1)	2.5 (1.6 - 3.3)
IES-R 7 ... I felt as if it hadn`t happened or wasn`t real.	2.5 (1.5 - 3.5)	1.4 (0.6 - 2.3)	1.8 (0.9 - 2.7)	1.3 (0.6 - 1.9)
IES-R 8 …I stayed away from reminders of it.	2.1 (1.1 - 3.0)	0.7 (0.1 - 1.3)	2.1 (0.9 - 3.2)	1.9 (1.1 - 2.6)
IES-R 9 … pictures about it popped into my mind.	3.2 (2.2 - 4.2)	1.9 (1.0 - 2.8)	3.2 (2.3 - 4.1)	2.6 (2.1 - 3.1)
IES-R 10 … I was jumpy and easily startled.	2.4 (1.5 - 3.3)	1.5 (0.7 - 2.3)	2.7 (1.3 - 4.0)	3.3 (2.3 - 4.4)
IES-R 11 … I tried not to think about it.	1.4 (0.6 - 2.2)	1.0 (0.3 - 1.7)	1.9 (1.0 - 2.8)	1.3 (0.6 - 1.9)
IES-R 12 …I was aware that I still had a lot of feelings about it, but I didn`t deal with them.	0.9 (0.2 - 1.6)	0.9 (0.3 - 1.6)	1.9 (0.9 - 2.9)	0.9 (0.3 - 1.4)
IES-R 13 … my feelings about it were kind of numb.	1.0 (0.3 - 1.7)	0.9 (0.1 - 1.6)	1.2 (0.3 - 2.1)	0.9 (0.3 - 1.6)
IES-R 14 … I found myself acting or feeling like I was back at that time.	2.0 (1.1 - 2.9)	0.8 (0.2 - 1.3)	1.5 (0.6 - 2.4)	1.4 (0.7 - 2.1)
IES-R 15 … I had trouble falling asleep.	2.8 (1.7 - 3.8)	1.1 (0.5 - 1.7)	3.7 (2.9 - 4.5)	3.8 (2.8 - 4.8)
IES-R 16 …I had waves of strong feelings about it.	2.8 (1.8 - 3.8)	0.9 (0.3 - 1.6)	2.5 (1.4 - 3.6)	2.5 (1.6 - 3.4)
IES-R 17 …I tried to remove it from my memory.	1.0 (0.3 - 1.7)	0.4 (-0.1 - 0.9)	1.3 (0.4 - 2.1)	0.9 (0.4 - 1.5)
IES-R 18 … I had trouble concentrating.	3.2 (2.3 - 4.0)	1.2 (0.6 - 1.9)	2.9 (1.6 - 4.1)	3.6 (2.5 - 4.7)

IES-R 19 … reminders of it caused me to have physical reactions, such as sweating, trouble breathing, nausea, or a pounding heart.	2.4 (1.4 - 3.5)	0.8 (0.3 - 1.4)	2.8 (1.5 - 4.1)	2.9 (1.7 - 4.0)
IES-R 20 … I had dreams about it.	1.9 (1.1 - 2.6)	1.1 (0.5 - 1.8)	1.7 (0.9 - 2.6)	1.5 (0.8 - 2.3)
IES-R 21 … I felt watchful and on- guard.	2.6 (1.6 - 3.7)	1.2 (0.5 - 1.9)	2.9 (1.8 - 3.9)	3.3 (2.2 - 4.5)
IES-R 22 … I tried not to talk about it.	1.1 (0.5 - 1.6)	0.4 (0.0 - 0.8)	1.7 (0.7 - 2.7)	1.5 (0.7 - 2.2)

CI = confidence interval. T1 = one week after birth. T2 = at infants' hospital discharge.

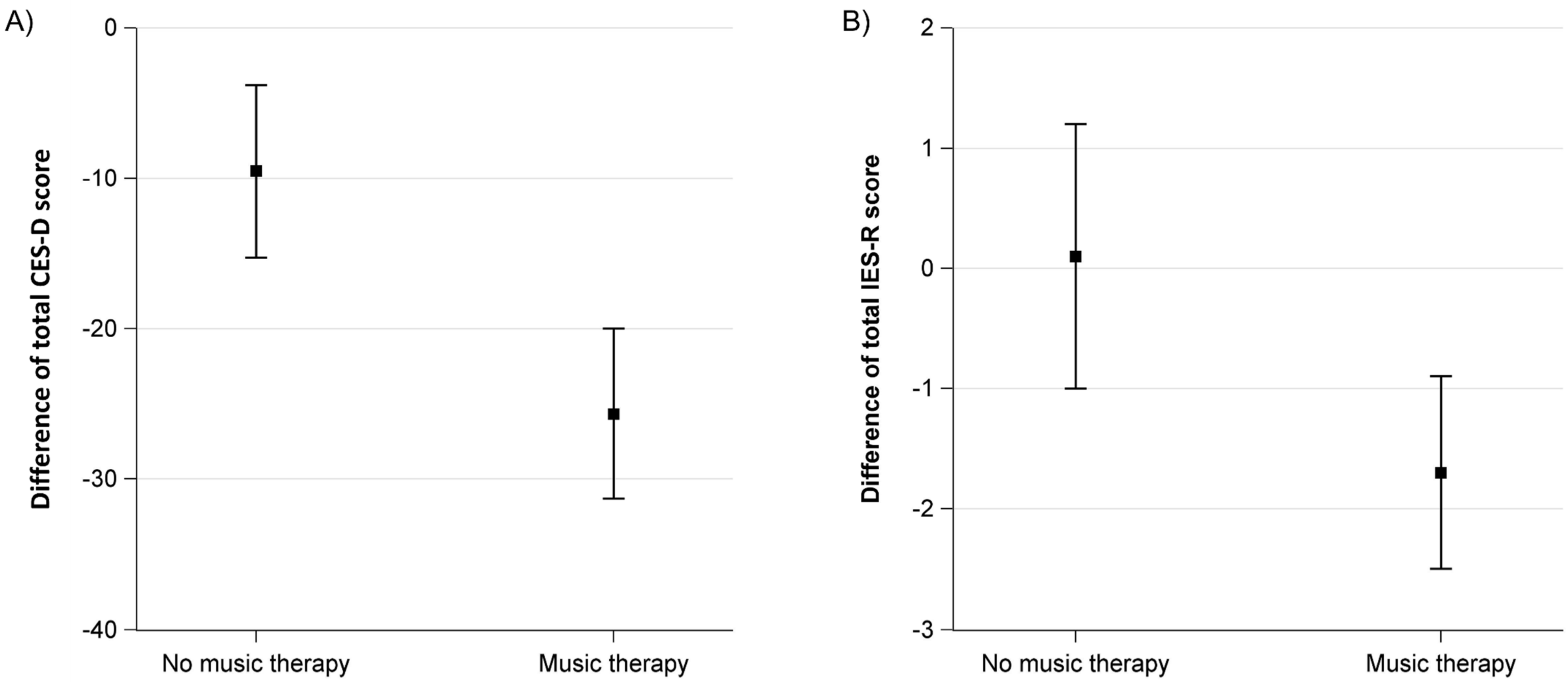

Figure S1: Mean differences of the total CES-D Scores and the total IES-R Scores of mothers of preterm infants born < 32 gestational weeks with and without music therapy between assessment one week after birth and the infant's discharge. (A) CES-D. (B) IES-R. Error bars represent 95 % confidence intervals.

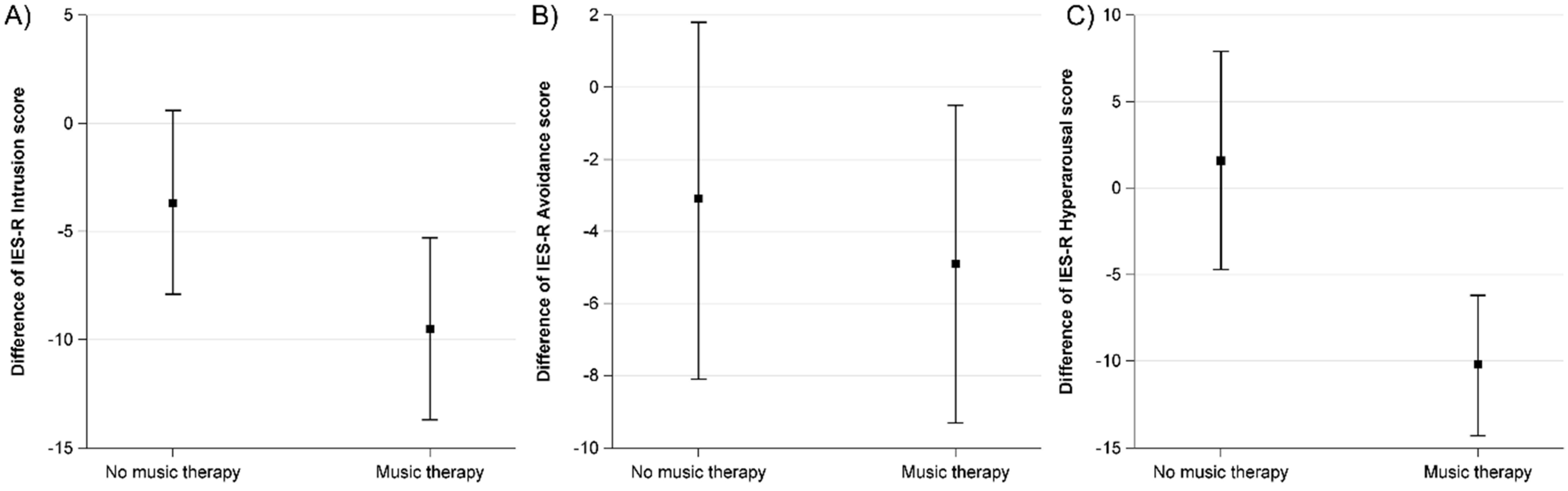

Figure S2: Mean differences of IES-R Items between mothers of preterm infant born < 32 gestational weeks with and without music therapy between assessment one week after birth and the infant's discharge. (A) Intrusion. (B) Avoidance. (C) Hyperarousal.
